# Peptide–protein docking: from physics-based models to generative intelligence

**DOI:** 10.1039/d6cc00583g

**Published:** 2026-03-18

**Authors:** Kai Ling, Shu Li, Zicong Zhang, Woong-Hee Shin, Daisuke Kihara

**Affiliations:** a Department of Computer Science, Purdue University, West Lafayette Indiana 47907 USA dkihara@purdue.edu; b Department of Biomedical Informatics, Korea University College of Medicine Seoul 02708 Republic of Korea; c Department of Biological Sciences, Purdue University, West Lafayette Indiana 47907 USA

## Abstract

Peptide–protein interactions (PepPIs) play a pivotal role in cellular signaling and regulation, representing a significant category of therapeutic agents. However, determining peptide–protein complex structures by experiment is costly and often challenging. Computational peptide–protein complex structure prediction, therefore, plays an important role in mapping binding modes and guiding design. Classical pipelines combine template-based, local, or global docking conformational search algorithms with physics-based or empirical scoring, but they often struggle with highly flexible peptides, induced fit at shallow interfaces, and non-canonical chemistries. In this review, we describe an ongoing shift from such conventional search-and-score workflows to deep learning-based pipelines. We categorize the modern methods into three modules: (i) approaches that predict likely peptide-binding regions on the protein surface and use these predictions to guide or filter docking models; (ii) AlphaFold-based protocols that use general structure prediction methods for peptide–protein co-folding and refinement; and (iii) deep generative models that sample peptide conformations given a target protein structure. We highlight that recent methods have substantially improved the accuracy and applicability of peptide–protein docking, while also identifying shared remaining challenges, including limited avaiability of training data and weak performance on long, disordered, or chemically modified peptides. We conclude by outlining directions for integrating richer biophysical constraints, better-curated peptide–protein datasets, and large-scale generative models to move toward robust, design-ready peptide docking.

## Abstract 2

We review the evolution of peptide–protein docking methods from traditional physics-based approaches to modern AlphaFold-inspired and diffusion-based frameworks. Their impact, remaining limitations, and open challenges are discussed.

## Graphical abstract

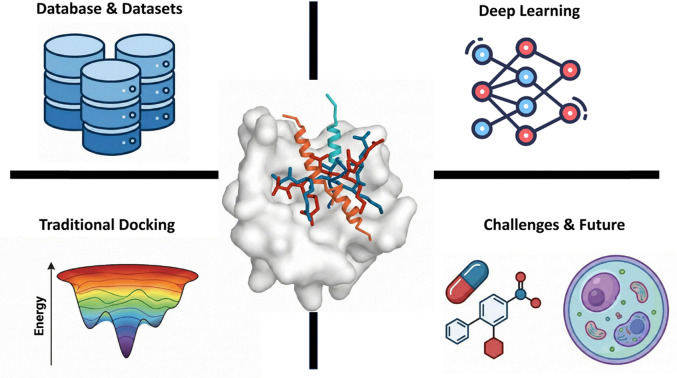

## Introduction

Protein–protein interactions (PPIs) regulate almost all cellular processes, including signal transduction, immune recognition, and metabolic control.^1,2^ Dysregulation of these interaction networks is associated with numerous diseases, such as cancer, metabolic disorders, and infectious diseases.^3,4^ Consequently, targeting PPIs has become an important strategy in modern drug discovery. However, conventional small-molecule drugs often struggle to modulate PPIs because many PPIs are large, flat, and lack well-defined binding pockets.^4,5^ Peptides may provide a promising alternative because their size and structural flexibility allow them to mimic natural interaction motifs and bind extended protein surfaces with high specificity.^6,7^ Many biological peptides function as signaling molecules or regulatory motifs that mediate transient peptide–protein interactions (PepPIs), which often control key cellular pathways.^7–12^ Understanding the structural and mechanistic principles of PepPIs is therefore essential both for elucidating cellular regulation and for developing peptide-based therapeutics.

In recent years, peptide therapeutics have emerged as an important drug modality for targeting PPIs and other biologically relevant protein interfaces.^7,10^ Approximately one hundred peptide drugs have been approved or are currently undergoing clinical trials worldwide.^13^ Compared to the traditional small-molecule drugs, peptides offer precise targeting capabilities required for protein complex interfaces, which are typically flat and difficult to target by small-molecule drugs.^14^ The modular architecture of peptide therapeutics also allows for systematic engineering to optimize binding affinity and selectivity. In addition, peptide drugs provide the ease of synthesis.

Another noteworthy application of PepPIs is in the context of masking peptides. The peptide, which is of a limited length, has been engineered to sterically block a functional binding site of a therapeutic molecule, such as an antibody. Consequently, it impedes the drug from interacting with its target while circulating in normal tissues. Subsequently, the blocking peptide is removed upon reaching the target cell, thereby enabling the therapeutic molecule to exert its activity at the target site.^15,16^ For instance, tumor-activated T-cell engager platforms, such as the TRACTr system developed by Janux Therapeutics, utilize peptide masks to impede CD3-binding domains until protease-mediated activation transpires in the tumor microenvironment.^17^

To develop peptide drugs or investigate PepPIs, it is important to obtain the three-dimensional structure of the complex. As with monomer structure characterization, experimental techniques such as X-ray crystallography, nuclear magnetic resonance, and cryogenic electron microscopy have been extensively utilized.^18^ Nevertheless, the experimental methods are characterized by their high resource demands and may face challenges in the context of transient complexes and membrane targets. It may also prove challenging to address the combinatorial diversity engendered by length variants and non-canonical amino acids solely through the utilization of experimental techniques. Therefore, computational peptide–protein docking methods can complement experimental approaches by providing atomic-level models of PepPIs.

In PepPI, a peptide has a length of 2 to 50 residues and usually binds to the larger protein. The problem could be translated into general protein–protein docking (PPD) by considering the peptide and the protein as ligand and receptor proteins, respectively. The field of PPD has established robust methodological foundations, such as the fast Fourier transform as used in ZDOCK^19^ and ClusPro,^20^ and geometric hashing as used in LZerD,^21^ which focuses on finding shape complementarity between the proteins. The PPD programs have demonstrated a degree of success in predicting PepPIs in certain cases. For example, Bonvin^22^ and his colleagues developed a protocol for cyclic peptide–protein complex structure prediction using their HADDOCK^23^ docking program. However, a limitation of these classical methods is that they treat the ligand as a rigid molecule to reduce the computational complexity, which might not be suitable for peptides, which are highly flexible. To address these complexities, classical docking strategies integrate global rigid body placement with conformational sampling and refinement, where the peptide is handled as a flexible molecule.^24–26^

More recently, the field of structure prediction has been revolutionized with the advent of deep learning methods such as AlphaFold,^27–29^ which have demonstrated a capacity for modeling monomer structures with high accuracy. Since then, deep learning based algorithms have been rapidly applied to predict protein complexes, such as AlphaFold-Multimer.^27^ However, direct transfer of such protocols to peptide docking has proven insufficient. Peptides are characterized by their intrinsic flexibility and small size, properties that enable them to fold upon binding and to form intricate hydrogen-bonding and electrostatic networks. Moreover, the fact that many peptides remain disordered in solution severely complicates conformational sampling. Given this rapid methodological evolution and the growing importance of peptides in drug discovery, this review surveys the development of peptide docking approaches from early PPD-based methods to cutting-edge deep learning frameworks and outlines future directions for achieving accurate, efficient, and biologically meaningful predictions of PepPIs. The methods discussed in this article are summarized in Table 1.

**Table 1:** Peptide–protein docking tools discussed in this article

Method	Year	Comments	Availability
Traditional docking methods			
Rosetta FlexPepDock	2011	High-resolution refinement protocol; couples peptide *ab initio* folding with docking refinement; requires approximate binding site.	https://flexpepdock.furmanlab.cs.huji.ac.il/
HADDOCK peptide docking	2013	Integrates conformational selection and induced fit.	https://www.bonvinlab.org/software/bpg/peptides/
pepATTRACT	2015	Fully blind coarse-grained peptide docking with two-stage atomistic refinement.	https://bioserv.rpbs.univ-paris-diderot.fr/services/pepATTRACT/
CABS-dock	2015	Fully flexible peptide docking without prior binding-site knowledge. coarse-grained sampling plus all-atom rebuilding.	https://biocomp.chem.uw.edu.pl/CABSdock
GalaxyPepDock	2015	Template-based protein–peptide docking using interaction similarity and energy-based optimization.	https://galaxy.seoklab.org/cgi-bin/submit.cgi?type=PEPDOCK
MDockPeP	2016	All-atom blind global peptide docking from peptide sequence and receptor structure using knowledge-based scoring.	https://zougrouptoolkit.missouri.edu/mdockpep/
PIPER-FlexPepDock	2017	Fragment-based global peptide docking with FFT sampling and high-resolution Rosetta refinement.	https://piperfpd.furmanlab.cs.huji.ac.il/
HPEPDOCK	2018	Blind hierarchical peptide–protein docking using an ensemble of peptide conformations.	https://huanglab.phys.hust.edu.cn/hpepdock/
AutoDock CrankPep (ADCP)	2019	Peptide-specific AutoDock engine. Folds and docks flexible, including cyclic peptides to a rigid receptor.	https://ccsb.scripps.edu/adcp/
PatchMAN	2023	Surface patch-matching. Threads peptide sequences onto structural motifs matched to the receptor surface, followed by refinement.	https://furmanlab.cs.huji.ac.il/patchman/
Site priors prediction methods			
PepSite	2012	Predicts peptide-binding spots on protein surfaces using statistical preferences. Does not generate peptide–protein complex structures.	https://pepsite2.russelllab.org
PepBind	2013	Sequence-based peptide-binding prediction framework that combines sequence and structure features. Does not generate peptide–protein docking structures.	https://yanglab.qd.sdu.edu.cn/PepBind/
PeptiMap	2017	Identifies potential peptide-binding sites on receptor surfaces by solvent-based surface mapping. Does not generate peptide–protein docking structures.	https://peptimap.cluspro.org
CAMP	2021	Sequence-based deep learning framework (CNN and self-attention) that jointly predicts peptide–protein interaction and peptide binding residues.	https://github.com/twopin/CAMP
PepNN	2022	Parallel structure-based and sequence-based models using reciprocal attention and graph neural networks to predict peptide-binding residues.	https://gitlab.com/oabdin/pepnn
PepCNN	2022	Deep learning predictor of peptide-binding residues that integrates sequence-based features and structural descriptors into a CNN.	https://github.com/abelavit/PepCNN
PepBCL	2022	Deep learning protein–peptide prediction. Used as a screening stage: scores docked peptide–target complexes to prioritize high-affinity binders for specific targets.	https://github.com/Ruheng-W/PepBCL
PepCA	2024	Sequence-based protein–peptide binding residue predictor built on protein language models and a cross-attention mechanism.	https://github.com/cloudaner115/PepCA
TpepPro	2025	Transformer-based peptide-binding site predictor that uses protein sequence representations and attention to identify peptide-binding regions on proteins.	https://github.com/wanglabhku/TPepPro
Pose reranking methods			
InterPepRank	2021	Graph convolutional network that encodes each docked peptide–protein decoy as a residue-level interaction graph and learns to re-rank poses.	https://bitbucket.org/isaakh94/interpeprank/src/master/
GraphPep	2025	Interaction-derived graph learning framework for scoring protein–peptide complexes. Uses graph neural networks/transformers to re-rank poses.	https://huanglab.phys.hust.edu.cn/GraphPep/
AF-based methods			
AF-Multimer w/Forced Sampling	2022	Peptide-specific protocol. Uses dropout/noise to generate diverse decoy ensembles for flexible peptides.	N/A
AF-augmented (Tsaban *et al.*)	2022	Uses AF2 with a poly-glycine linker to model peptide-protein interactions.	N/A
MHC specific AF			
(Motmaen *et al.*)	2023	Fine-tunes AF by adding a classifier head and jointly optimizing binding classification and structure prediction. Applied on peptide–MHC interactions.	https://github.com/phbradley/alphafold_finetune
MHC-Fine	2024	Fine-tuned AF model trained exclusively on high-resolution MHC–peptide crystal structures.	https://bitbucket.org/abc-group/mhc-fine/src/main/
DistPepFold	2025	Knowledge distillation method. Improves AFM by training teacher and student models.	https://github.com/kiharalab/DistPepFold
Diffusion based methods			
RAPiDock	2025	SE(3)-equivariant diffusion model. Uses bi-scale graphs and physical constraints for rapid, all-atom peptide docking.	https://github.com/huifengzhao/RAPiDock
DiffPepDock	2025	Diffusion model trained on synthetic fragments as initial training.	https://github.com/YuzheWangPKU/DiffPepBuilder

## Conventional docking methods

Traditional peptide–protein docking evolved from geometric and energetic frameworks, originally designed for protein–protein interactions. It was adapted to accommodate greater flexibility and a smaller size of peptides. In practice, these tools generally fall into three categories based on their scope:^30^ Template-based methods, methods that perform local refinement, and global docking. These three categories differ primarily in the amount of prior knowledge required. Template-based methods rely on previously determined structures of similar protein–peptide complexes, which are used as scaffolds for modeling. Local refinement methods assume partial knowledge of the target complex, such as a predefined binding site or known contact residues, to restrict the search space. In contrast, global docking methods perform peptide position and pose sampling over the entire receptor surface without prior specification of the binding region.

Template- and motif-based methods, such as GalaxyPepDock^31^ and PatchMAN,^32^ transfer interaction patterns or surface-bound peptide backbones from known structures to new targets. They achieve this either by identifying proteins with highly similar sequences or by matching local shapes on the receptor surface using large structural databases. GalaxyPepDock is highly accurate when structurally similar protein–peptide complexes are available in existing databases. On the other hand, the main limitation of this approach is its strict dependence on the availability of suitable templates. Consequently, the method typically fails when predicting entirely novel binding modes or when targeting proteins with no known structurally similar references. On the other hand, PatchMAN does not require complete complex templates to operate. Instead, it identifies small structural fragments from known proteins that are complementary to the receptor surface. While the system is capable of handling non-standard amino acids with post-translational modifications, a key limitation is that its initial search relies on rigid sampling. Lastly, it may struggle to successfully place and refine peptides in cases where a “closed” receptor pocket creates severe steric clashes with the peptide backbone.^32^

When the binding site is already defined, local refinement protocols are a standard choice. Methods such as Rosetta FlexPepDock,^33^ HADDOCK peptide docking,^26^ and even adapted small-molecule engines such as AutoDock Vina^34^ operate by restricting conformational sampling to a specific box. Rosetta FlexPepDock is reported to generate accurate models by allowing full flexibility of the peptide and the receptor side chains.^24,35^ Its primary disadvantage is its inability to perform fully blind docking. Also, a key limitation is its reliance on an accurate starting position, and the receptor backbone remains largely rigid. HADDOCK offers a significant advantage when biochemical or biophysical experimental data are available, which are used as constraints.^36^ Conversely, the accuracy of the method strongly depends on the availability of experimental restraints. AutoDock Vina is fast and uses an efficient empirical scoring function. However, its limitation is that it cannot handle compounds with too many rotatable bonds, *i.e.*, long peptides.^37^

Global or “blind” docking presents a different challenge: tools such as HPEPDOCK,^38^ PIPER–FlexPepDock,^24^ and CABS-dock^25^ navigate the entire receptor surface to identify the peptide binding site. To handle this large computational load, they typically rely on coarse-grained models or staged searches. MDockPeP^39–41^ works in three steps: first, it creates multiple peptide backbone conformations. Then, it docks each conformation independently on the whole receptor surface using a method based on AutoDock Vina. Finally, docked peptides are ranked using a scoring system specific to protein–peptide interactions. HPEPDOCK is a fast global docking method that represents peptide flexibility using multiple pre-generated conformations, avoiding costly sampling during docking. Because these conformations are generated without consideration of the receptor and treated as rigid, the method is less accurate when binding requires large structural changes and for long peptides with large conformational space. PIPER-FlexPepDock combines global docking with high-resolution refinement, enabling peptide flexibility and receptor side-chain adjustment. However, the method is computationally expensive, and the rigid-receptor assumption in the initial search limits performance when backbone rearrangement is required. CABS-dock uses a coarse-grained model for blind docking, enabling high flexibility of both partners without prior knowledge,^23^ but the simplified representation may reduce accuracy, particularly for interactions requiring detailed atomic contacts. MDockPeP is an efficient *ab initio* docking method that performs fully blind global docking using only the receptor structure and peptide sequence. It combines template-based peptide modeling with global rigid sampling and local flexible refinement, making it faster than methods based on molecular dynamics. However, the receptor is treated as rigid, and accuracy decreases for long peptides. IDP-LZerD tackled the docking of long disordered peptides by docking fragments of disordered peptides and assembling them into the full chain length.^42,43^ Although it provided a novel idea, the accuracy of long peptides is limited.

These classical approaches share a common architectural pipeline. The process begins by generating a large number of initial ensembles of peptide poses. While template-based methods derive backbone conformation from structurally similar complexes, local and global schemes typically employ rigid-body sampling, fragment assembly, or coarse-grained models to populate the receptor's surface with candidate orientations. Subsequently, scoring functions that evaluate coarse-grained features of the models are used to screen candidate conformations, for example, by assessing shape complementarity and physicochemical properties. Selected candidates often undergo a high-resolution refinement stage at all-atom resolution, allowing adjustments of the peptide backbone and limited flexibility of the receptor.

The ability of classical docking to recover near-native poses hinges largely on the efficacy of scoring and refinement. Primary scoring functions vary widely in their approaches: they range from knowledge-based statistical potentials (*e.g.*, ITScorePeP in MDockPeP^39^) and hybrid energies (GalaxyPepDock^31^) to the coarse-grained interaction terms found in pepATTRACT^44^ or CABS-dock.^25^ At the other end of the spectrum lie physics-based functions, such as those used by Rosetta FlexPepDock^33^ and the AutoDock^45^ family, including ADCP,^35,46^ which evaluate interactions on pre-calculated affinity grids. All these functions face the difficult task of distinguishing native-like interfaces from a vast sea of decoys while remaining computationally efficient. To address the limitations of fast scoring, refinement stages apply high-resolution, all-atom energy functions to a narrowed candidate set. For instance, FlexPepDock^33^ employs intensive Monte Carlo moves and gradient minimization in an all-atom potential, whereas pepATTRACT^44^ and CABS-dock^25^ transition from simplified coarse-grained docking to high-resolution atomistic reconstruction. Earlier protein–protein docking frameworks, such as LZerD,^21,47^ which combine geometric shape descriptors with energy-based filtering, helped establish scoring and refinement ideas that modern peptide docking engines adapt to flexible ligands.

Classical geometric docking suffers from three main limitations. Combinatorial growth of the search space with peptide length and flexibility prevents exhaustive sampling, especially for long peptides. Rigid receptor backbones fail to capture loop motions or order–disorder transitions, making results highly dependent on the input conformer. Reliance on binding-site priors, such as experimental data, adds fragility, as inaccurate or missing site information produces models that cannot be reliably ranked. These challenges reflect the static-energy landscape assumption, which deep learning-based approaches aim to overcome.

In the last paragraph in this section, we mention metrics used to evaluate the accuracy of docking models. Commonly, model evaluation follows metrics used in the community-wide evaluation of protein docking, the Critical Assessment of Predicted Interactions (CAPRI).^48^ The CAPRI criteria evaluate models using three metrics: the root mean square deviation (RMSD) of the ligands (L-RMSD), RMSD of interface atoms (iRMSD), and the fraction of native contacts (fnat). The DockQ^49^ score, which combines these three metrics, is also frequently used lately. Additional geometric and stereochemical evaluations, such as Ramachandran plot statistics,^50^ rotamer outlier analysis,^51^ MolProbity score,^52^ and buried surface area assessment,^53^ are also often used.

## Recent evolution of peptide–protein docking methods

**Fig. 1:**
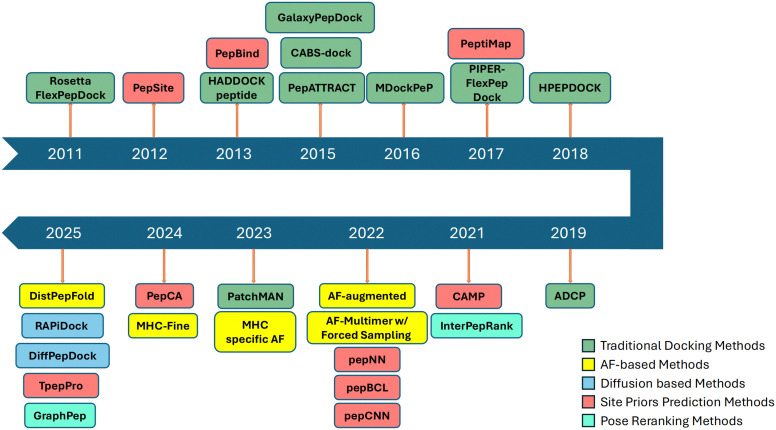
Timeline of peptide-docking methods from 2011 to 2025, grouped by conceptual class. Methods mentioned in this work are classified into five categories. Early work is dominated by traditional search-and-score docking engines (green). From 2021 onward, deep learning-based frameworks emerge, including AF-based models (yellow) and diffusion-based models (cyan). In parallel, two auxiliary components, site-prior (binding site) prediction methods (red) and learned pose re-ranking strategies (mint).

Peptide–protein docking has progressed substantially from its early approaches mentioned above. The evolution of method development is schematically summarized in Fig. 1. Deep learning began to be introduced into key components of this problem around 2012,^54–57^ with a marked increase in such studies after approximately 2020, coinciding with the introduction of AlphaFold2^28^ into the field. In protein–peptide docking, two categories of auxiliary methods that complement complex structure modeling have been actively pursued: methods that predict peptide-binding sites on receptor proteins and methods that rank docking models. Since 2022, nearly all newly proposed approaches have incorporated deep learning components. Most recently, we have observed methods that use diffusion models, a generative deep learning approach.

### Peptide binding site prediction and docking pose re-ranking methods

There have been notable efforts to develop methods for predicting peptide binding sites. These methods predict where on the receptor surface a peptide is likely to bind, at residue or patch resolution. Predicted binding sites can be used in the downstream docking, co-folding, or in generative engines as masks, spatial constraints, or re-ranking signals.^23,33,39,58–60^ These methods use hand-crafted physicochemical, geometric, or statistical features rather than learned representations. PepSite^59^ and PeptiMap^60^ identify peptide-binding patches on protein surfaces by detecting statistically meaningful residue–residue contact patterns or energetically favorable fragment placements. PepBind^58^ infers peptide-binding regions from sequence-derived features, including residue composition and evolutionary profiles that are integrated in a support vector machine, a type of machine learning algorithm, and integrates these predictions with binding-site annotations transferred from structurally similar proteins in the BioLiP database.^61^

From around 2020, we observed the appearance of methods using deep learning. PepNN-Struct/Seq,^62^ Pep-CNN,^63^ and PepCA^64^ exploit receptor geometry, sequence context, and local physicochemical features to highlight peptide-binding hotspots. PepNN-Struct models the receptor as a residue-level graph derived from the protein structure and applies attention mechanisms to predict peptide-binding residues. Pep-CNN and PepCA primarily operate on residue-level representations derived from sequence and local physicochemical features. Pep-CNN employs convolutional neural networks on residue-wise feature maps combining evolutionary and structural descriptors, whereas PepCA uses protein language model embeddings together with co-attention mechanisms to infer residue-level peptide-binding sites.

There are also methods that predict peptide binding residues using sequence information. CAMP^65^ is a multi-level framework that takes sequence-derived feature profiles of both peptide and protein as input, predicting whether a given protein–peptide pair interacts and, additionally, identifying binding residues along the peptide sequence. TPepPro^66^ is a Transformer-based PepPI predictor that integrates local sequence representations of proteins with global structural context derived from protein contact maps, and outputs pair-level interaction likelihoods rather than explicit binding sites. PepBCL^67^ uses the BERT language model as the base. It uses sequence information of a protein and a peptide only and uses contrastive learning to predict protein-side peptide-binding residues at the residue level.

In the majority of computational pipelines, binding site prediction is not treated as an endpoint; rather, it functions as a modular component that facilitates the conversion of blind global searches into constrained quasi-local exploration. Additionally, they contribute to the stabilization of pose generation and ranking when experimental or template-derived site information is incomplete or noisy.

Peptide docking pose re-ranking is another important component in peptide docking. After generating an initial ensemble of peptide poses, a challenge is to reliably identify which decoys (generated models) are worth refining. Early work approached this by examining if predicted peptide poses overlap with a designated binding site. For example, decoys were re-ranked based on the overlap between predicted or restrained binding interfaces and the interfaces formed in docking models.^23^ Other approaches employed knowledge-based statistical potentials derived from known peptide–protein complexes (*e.g.*, ITScorePeP in MDockPeP^39^). In addition, consensus- and clustering-based strategies prioritized decoys belonging to large, densely populated interface clusters under the assumption that near-native binding modes are sampled repeatedly, as implemented in peptide docking frameworks such as CABS-dock,^25^ pepATTRACT,^44^ and the PIPER–FlexPepDock^24^ pipeline.

More recent methods learn re-ranking from decoy ensembles using deep learning. InterPepRank^68^ encodes each peptide–protein decoy as a residue-level graph with physical contacts as edges and trains a graph convolutional network to predict model quality, which substantially improves the selection of starting structures for FlexPepDock^33^ refinement. GraphPep^69^ takes a more interaction-centric view, representing decoys as graphs whose nodes correspond to protein–peptide contacts rather than individual residues, and learns to distinguish near-native from incorrect poses using a mixture of classical docking and AlphaFold-generated^27–29^ decoys. In both cases, supervision is provided by continuous or discretized quality labels such as DockQ^49^ docking score or CAPRI prediction accuracy classes,^48^ and models are typically optimized with regression or ranking losses.

### AlphaFold-based peptide docking methods

After AlphaFold2 (AF2) was developed, Tsaban *et al.*^70^ extended the capabilities of AF2, which is fundamentally a protein monomer prediction framework, by introducing modified inputs that enable the modeling of protein–peptide complexes. This approach connects receptor sequence and peptide sequence with a poly-glycine linker. It was shown that without further training, AF2 is able to model protein–peptide complexes. Similarly, Motmaen *et al.*^71^ extended AF2 to jointly predict protein structure and major histocompatibility complex (MHC)-peptide-binding specificity, where MHC and peptide sequences were connected and used as input to AF2. However, caution is required when using AF2 for peptide docking, as peptide predictions by AF2 have been shown to be biased by the peptide's secondary structure.^72^

Shortly after AF2, AlphaFold-Multimer (AFM)^27^ was developed for modeling protein complexes. Even though AFM has demonstrated high accuracy in modeling general protein complexes, it has shown that the accuracy of modeling protein–peptide complexes lags behind that for single-chain proteins and regular protein complexes.^70,73–77^ Johansson-Åkhe *et al.*^74^ reported that increasing the recycle number and generating more structures in AFM can lead to better modeling performance. The approaches by Tsaban *et al.*^70^ and Johansson-Åkhe *et al.*^74^ preserve the original AFM architecture, avoid additional training cost, and maintain broad applicability, but they are inherently limited by the capacity of AFM for peptide docking. MHC-Fine improved prediction accuracy for MHC–peptide complex structures by further training AFM with larger neural networks and adding protein–peptide interaction information to templates during training.^78^ Phospho-Tune improved structure modeling of phosphorylated peptide and protein interactions by further training AFM specifically on phosphopeptide–protein complexes with an integration of phosphorylation information, such as the position of phosphorylated residues in the peptide sequence, during the training process.^79^

MHC-Fine and Phospho-Tune are examples of methods that target specific biological applications. Limitations could be the size and diversity of available datasets for these specific applications. The relatively small number of known peptide–protein complex structures constrains a comprehensive assessment of model robustness. Broader and more diverse benchmarks are desired to validate the stability and reliability of models fine-tuned for specific peptide–protein interactions, but it is actually a challenge common to all types of current peptide-docking methods.

### DistPepFold: improving peptide–protein docking using privileged knowledge distillation

**Fig. 2:**
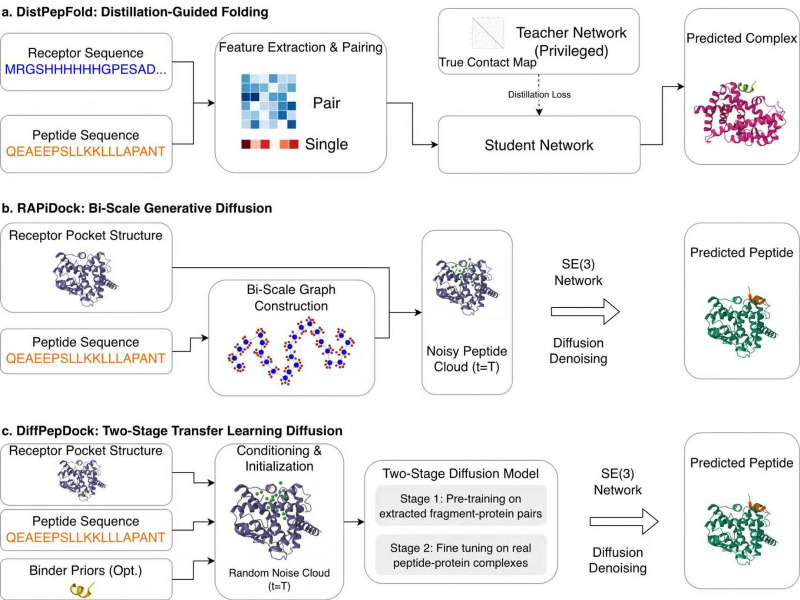
Workflows of DistpepFold, RAPiDock, and DiffPepDock. The diagrams illustrate the workflows of the three methods. See text for explanation. (a) DistPepFold; (b) RAPiDock; (c), DiffPepDock.

DistPepFold^73^ improves protein–peptide docking by applying a privileged knowledge distillation approach. Fig. 2a shows the workflow of the algorithm. DistPepFold uses a teacher-student framework, where two models, the teacher model and the student model, are trained. The teacher model is trained first with privileged knowledge, which in this case is the set of interacting residue pairs between the receptor and the peptide in this case. With this critical information on interacting residues, the teacher model achieved high modeling accuracy. The idea of the student-teacher framework is to indirectly transfer the privileged knowledge to the student model through the training process. During the distillation process, the student model is instructed to mimic the behavior of the teacher model, or more concretely, reproduce intermediate outputs of the teacher model, in addition to the correct complex model for the target. During the inference stage, only the student model is used for predicting structures. DistPepFold outperforms AFM and other existing methods in terms of several metrics, where DistPepFold predicted more high-quality structures evaluated using the peptide docking accuracy criteria used in the CAPRI docking competition.^48^ DistPepFold is available on GitHub at https://github.com/kiharalab/DistPepFold.git.

**Fig. 3:**
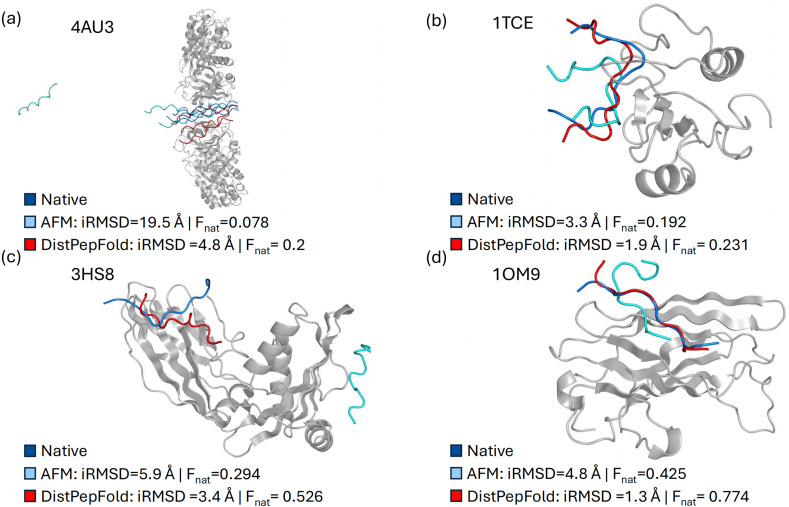
Examples of predicted protein–peptide complex structures. AlphaFold-Multimer predictions (cyan) and DistPepFold predictions (red) are shown with the native peptide conformations (marine). The receptor structure is rendered in a grey cartoon. We did not show predicted receptor structures as they were close to the reference structure in the PDB database. (a) Procollagen-specific molecular chaperone complexed with Collagen model peptide 15-R8 (PDB ID: 4AU3). The complex consists of two receptors of 392 residues and three peptides of 20 residues. (b) Complex of the SHC SH2 domain and tyrosine-phosphorylated peptide (PDB ID: 1TCE). The protein–peptide complex consists of a receptor of 107 residues and a peptide of 20 residues. (c) Adaptor protein complex AP-2 with peptide from intersectin-1 (PDB ID: 3HS8). The protein–peptide complex consists of a receptor of 273 residues and a peptide of 12 residues. (d) ADP-ribosylation factor binding protein GGA1 with the 15-mer peptide fragment of p56 (PDB: 1OM9). The protein–peptide complex consists of a receptor of 154 residues and a peptide of 15 residues. For each method, interface RMSD (iRMSD, Å) and Fnat scores are reported. iRMSD quantifies the root-mean-square deviations of interface residues between predicted and native peptide conformations after receptor superposition. Fnat represents the fraction of native interfacial contacts recovered in the structure model, which ranges from 0 to 1 (the best). According to CAPRI criteria for protein–peptide docking, iRMSD cutoffs of >2.0/2.0–1.0/1.0–0.5/<0.5 Å and Fnat cutoffs of <0.2/0.2–0.5/0.5–0.8/≥0.8 correspond to incorrect/acceptable/medium/high-quality models, respectively.

In Fig. 3, four examples of structure models predicted by DistPepFold are shown in comparison with AFM. The models shown here are generated by the student model, as the teacher model needs correct residue contact information, which is not available in an actual prediction scenario. We did not show predicted receptor structures as they were close to the reference structure in the PDB database.^80^ For these examples, we showed two metrics, interface root-mean square deviation (RMSD; iRMSD) and the fraction of the native contacts (Fnat). iRMSD quantifies the deviation of residue positions at the binding interface in the model relative to the reference structure in PDB. Fnat measures the proportion of native residue–residue contacts that are correctly recovered in the model.

In the first example (Fig. 3a), AFM failed to identify the peptide binding site, causing the peptide to detach from the receptor structure. In contrast, DistPepFold successfully identified a near-native pose of the bound peptide, resulting in a substantial improvement in iRMSD from 19.5 Å to 4.8 Å. In the second example (Fig. 3b), AFM predicted the correct peptide binding site, but the peptide conformation was incorrect. DistPepFold correctly identified the binding site and produced a peptide conformation close to the reference PDB structure. In the third example (Fig. 3c), AFM docked the peptide at an entirely incorrect location (cyan), whereas DistPepFold accurately identified the correct binding site. In the final example (Fig. 3d), AFM predicted a slightly shifted peptide binding site, resulting in the loss of many native contacts. By contrast, DistPepFold predicted the correct binding pose at the correct site. This improvement is reflected in the iRMSD values (4.8 Å for AFM and 1.3 Å for DistPepFold).

### Generative peptide docking using the diffusion model

Recent advances in peptide docking utilize diffusion models, adapting deep generative strategies that have revolutionized fields such as computer vision and structural biology. The diffusion model has been used as the core of the structure generation engine of recent protein and DNA/RNA structure prediction methods. A representative method in this category is AlphaFold3,^29^ and there are other similar methods with a diffusion model, including RoseTTAFold All-Atom,^81^ HelixFold-Multimer,^82^ Boltz-1,^83^ Chai-1,^84^ and ProteniX.^85^

The diffusion model was also introduced in protein–peptide docking to overcome the physical complexity and data scarcity inherent in peptide docking. In RAPiDock^86^ (Fig. 2b), instead of moving atoms individually, which can lead to distorted structures, a step-by-step diffusion process is employed that manipulates the peptide only through physically valid degrees of freedom: global rotation, translation, and torsion angles. To achieve computational efficiency without sacrificing resolution, the model represents peptides using a graph with two types of nodes, coarse-grained residues for rapid global placement and fine-grained atoms for precise side-chain modeling. Furthermore, it uses Clebsch–Gordan tensor products^87^ to provide a rigorous mathematical framework for enforcing SE(3)-equivariance. This ensures that the model inherently respects rotational symmetry, allowing it to achieve high-throughput screening speeds while maintaining atomic-level precision. Another method, DiffPepDock^88^ (Fig. 2c), tackles the scarcity of data on peptide–protein complex structures. This method uses a two-stage training strategy. First, the network is pretrained using a dataset of protein-fragment complexes extracted from protein–protein complexes, which mimic protein–peptide interactions. Extracted fragment-protein complexes are approximately four times more abundant than available peptide–protein complexes. In the second stage of training, the network is fine-tuned on real peptide–protein complexes.

Collectively, representative diffusion-based peptide docking methods and general-purpose structure predictors exhibit complementary strengths and characteristic limitations. Specialized docking frameworks implemented in RAPiDock and DiffPepDock provide high sampling efficiency and strong docking accuracy, particularly for short to medium-length peptides binding to relatively rigid receptors. Their physically constrained diffusion processes reduce structural distortions and improve pose generation. However, they may underperform when substantial receptor conformational changes occur, or when binding modes are insufficiently represented in the training data. In contrast, general-purpose structure predictors, including AlphaFold 3, ProteniX, RoseTTAFold All-Atom, HelixFold-Multimer, Boltz-1, Boltz-2, and Chai-1, provide broad applicability across diverse biomolecular complexes but are not specifically optimized for peptide docking. Moreover, these models rely heavily on multiple sequence alignments (MSAs) and evolutionary information to infer inter-chain contacts, and their performance may decrease for short peptides or orphan sequences with a limited MSA depth. They may also misidentify binding sites or mis-rank alternative poses when evolutionary signals are weak.

Finally, it is worth mentioning that the diffusion model is also used in the *de novo* design of peptides that bind at the target pocket. Methods in this category design the binding peptide sequence instead of docking a known peptide to a receptor structure. The pipeline of RFdiffusion^89^ and ProteinMPNN^90^ is a representative workflow where the former designs a structure of the protein or peptides that meet the provided condition (*e.g.*, binding to a pocket in a receptor protein) and the latter designs amino acid sequences that fold into the designed structure. This paradigm is rapidly expanding with other binder design frameworks such as ODesign,^91^ BoltzGen,^92^ and BindCraft.^93^ ODesign and BoltzGen generalize this process by performing simultaneous co-design of both sequence and structure within unified all-atom generative models, enabling multimodal and flexible conditioning. BindCraft leverages AlphaFold-based hallucination and gradient-based optimization to iteratively refine binder sequences and interfaces, emphasizing one-shot functional binder generation rather than diffusion-based backbone sampling. While the resulting protein–peptide complex is structurally indistinguishable from a docked pose, these methods fundamentally operate by solving the inverse folding problem through generative design, rather than stochastic sampling of a pre-existing sequence.

## Challenges and future directions of the field

Peptide–protein docking methods have evolved markedly over the past decades. Early approaches largely adapted rigid-body protein–protein docking frameworks, extending them with coarse-grained sampling, fragment-based assembly, and local refinement to accommodate peptide flexibility. These classical methods established practical baselines but were limited by the size of the conformational search space they could explore and the accuracy of heuristic scoring functions. The introduction of deep learning enabled data-driven modeling of peptide–protein interactions, improving binding-site identification and pose selection. More recently, generative models, including diffusion-based approaches, have advanced peptide docking by learning conditional distributions over peptide–protein complex conformations rather than relying on exhaustive search-and-score sampling. Recent methods report substantial improvement in the modeling accuracy over earlier methods. For example, DiffPepDock,^88^ a diffusion-based peptide docking method, reported that the mean ligand RMSD (RMSD of a predicted ligand pose relative to the native when the receptor structure is superimposed) ranged from ∼11 Å for classical docking methods to ∼4.5 Å. Similarly, RAPiDock^86^ achieves over 50% success for high-quality predictions under CAPRI criteria among top-100 ranked models, compared with ∼20–30% for classical docking baselines.

Despite substantial progress, peptide–protein docking methods remain less accurate than single-chain protein structure prediction, reflecting several methodological limitations. A major challenge is the rapid growth of conformational space with peptide length and flexibility, which prevents exhaustive sampling of long or macrocyclic peptides within a realistic computational time. Deep learning-based approaches improve accuracy but depend heavily on patterns learned from existing structural datasets, performing well on common interface types but generalizing poorly to long, disordered, or chemically modified peptides underrepresented in training data. Many end-to-end models treat docking primarily as a structure prediction task rather than an explicit binding process, limiting their ability to model alternative binding modes or conformational ensembles. As a result, different methods succeed under specific assumptions but fail outside their intended regimes, highlighting the need for approaches that balance sampling, physical realism, and reliable scoring. A further unresolved issue is the reliability of confidence estimation.^94,95^ While many modern pipelines provide confidence scores or ranking metrics, these scores are often poorly calibrated with respect to true docking accuracy, particularly for chemically diverse peptides or alternative binding modes. Improving the interpretability and calibration of confidence estimates remains essential for translating peptide docking predictions into practical decision-making.

Looking forward, peptide–protein docking is likely to continue making incremental progress through the adoption of increasingly advanced deep learning techniques emerging from the machine learning community. However, achieving fundamental improvements will require the construction of new, high-quality datasets. In particular, the development of experimentally determined peptide–receptor complex structures and associated energetic measurements, as a community-wide effort, would provide a critical foundation for next-generation methods. Expanding training and benchmark datasets to better represent long, cyclic, and chemically modified peptides will also be essential for improving performance beyond the current set of curated cases. In parallel, integrating physically motivated energetic models with deep learning may help compensate for limited training data and enable more principled generalization. Together, these advances will be necessary to move peptide–protein docking from incremental gains toward robust, broadly applicable predictive modeling.

## Author contributions

DK conceived the study. KL, SL, and ZZ drafted the manuscript. WHS and DK critically edited it. All the authors read and approved the manuscript.

## Conflicts of interest

There are no conflicts to declare.

## Data Availability

This article is a review, and no new data is produced.
